# An impaired learning environment: Resident physicians’ experience of the transition to pandemic care during the first wave of the COVID-19 pandemic in Sweden

**DOI:** 10.3389/fpsyg.2022.1090515

**Published:** 2023-01-06

**Authors:** Emma Brulin, Kristina Henriksson, Bodil J. Landstad

**Affiliations:** ^1^The Department of Occupational Medicine, Institute of Environmental Medicine, Karolinska Institutet, Stockholm, Sweden; ^2^Unit of Paediatrics, Östersund Hospital, Östersund, Sweden; ^3^The Department of Health Sciences, Mid Sweden University, Östersund, Sweden; ^4^The Unit of Research, Education and Development, Östersund Hospital, Östersund, Sweden

**Keywords:** COVID-19, resident physician, learning environment, clinical work, experiences, Sweden

## Abstract

**Introduction:**

Extensive studies regarding the COVID-19 pandemic have shown negative effects on physicians-in-training. Besides a high workload, their learning environment has been affected. A quality learning environment is vital for residents’ physician’s clinical development and also their health. Nevertheless, few studies have explored this. The aim of this study was to explore resident physicians’ experiences of transition to pandemic care during the first wave of the COVID-19 pandemic in Sweden.

**Method:**

In this qualitative study, 12 Swedish resident physicians were interviewed using a semi-structured interview guide. They were interviewed between June and October of 2020 and asked to reflect on the pandemic and, more specifically, the first wave. The empirical material was analysed using qualitative content analysis. The analysis resulted in one theme and four categories.

**Results:**

The theme identified was *An impaired learning environment* which signifies the disruptions the resident physicians experienced during the first wave of the pandemic. The four categories, Professional role insecurity, High expectations but little influence, Stagnant clinical development, and Professional growth through experience, describe in what way the learning environment was impacted.

## 1. Background

The COVID-19 pandemic has globally had a high clinical impact. Many physicians experienced extreme workload (Eftekhar Ardebili et al., 2021), lack of support (Billings et al., 2021) and poor management (Mohammadi et al., 2021), which adversely affected physicians’ mental health (De Sio et al., 2020; Leo et al., 2021; Hagqvist et al., 2022). In Sweden, the negative health effect was more significant among physicians-in-training than senior physicians (Hagqvist et al., 2022). The COVID-19 pandemic did not only impact the work environment of resident physicians, i.e., physicians under supervised training to get a certificate of specialist expertise but severely affected the clinical learning environment for resident physicians (Dedeilia et al., 2020; Chen et al., 2021). Essential aspects of the learning environment include supervisory support, accessibility to supervisors, teamwork (e.g., peers, nurses, and other hospital personnel), mutually supportive and beneficial relationships with supervisors (Roff et al., 2005), a good work environment, and reasonable working hours (Ironside et al., 2019). However, a recent review shows that the most commonly reported effect of the pandemic on residents learning environment was decreased clinical experience and failure to meet the training requirements of the medical specialty (Chen et al., 2021). More inexperienced residents rated the supervision as poor and inadequate (Young et al., 2022). Practical elements of the specialist training, i.e., educational activities, were cancelled, and there has been a transition towards digital learning (Alam et al., 2021; Wådell et al., 2022). Time on hands-on-training in operating specialities was lost and cannot be compensated for (Wådell et al., 2022). Excessive work hours, heavy workloads, transfer to COVID-ward, and moral dilemmas have also been reported among residents (Chen et al., 2021; Farrell and Hayward, 2022; Wådell et al., 2022).

The learning environment for resident physicians is vital, not only for their professional development but for their health and well-being. The COVID-19 pandemic has significantly increased psychological distress and burnout among physicians (De Sio et al., 2020; Leo et al., 2021), not the least among resident physicians (Kaplan et al., 2021; Moini et al., 2021; Hagqvist et al., 2022). While a functioning and high-quality learning environment can play a pivotal role in preventing burnout among resident physicians (Ironside et al., 2019), it can be detrimental when quality is low (Dyrbye and Shanafelt, 2016; van Vendeloo et al., 2018; Lu et al., 2021). Factors in resident physicians‘learning environment that can contribute to poor mental health have been shown to be a lack of collegial support, poor transparency, experiences of unfair decision-making by healthcare management (Mihailescu and Neiterman, 2019), high demands on education and clinical or professional development, lack of independence (Dyrbye and Shanafelt, 2016), and substandard supervision (Dyrbye et al., 2018). Moreover, when there is an imbalance between training and clinical responsibility, there is an increased risk of burnout (Lu et al., 2021). On the other hand, burnout and stress can, in turn, contribute to reduced motivation for education and clinical development (Dyrbye et al., 2018; Lu et al., 2021).

Resident physicians in Sweden are required to fulfil the learning objectives for each specialty as specified in the regulations on doctors’ medical specialty training by the Swedish national board of health and welfare (SOSFS 2015:8). These required learning objectives include supervised clinical work corresponding to a minimum of 5 years of full-time work, specialist expertise courses, and external residency-terms. Thus, training, courses and supervision are central to becoming an independent attending physician with specialist competence. Statistics from the Swedish Medical Association show that as many as 69% of their member associations lag with courses and certification for resident physicians because of the pandemic (Hjelmqvist and Johansson, 2021). There is also an expressed concern among several specialist associations in Sweden that the specialist training for most resident physicians will need to be extended because of cancelled training and learning sessions (Pagels, 2021). Among OB-GYN residents in the south of Sweden, as many as 95% of the residents report an impact on their specialist training (Wådell et al., 2022).

Although the COVID-19 pandemic seemed to have had adverse effects on resident physicians’ work and learning environment, few studies have explored their experiences of the environment in which they train and work. Among the studies that were found, most have focused on quantitative outcomes rather than the experiences of the residents (Chen et al., 2021). Knowledge regarding how physicians experienced the pandemic is vital to minimise the impact on resident physicians in future crises by giving them the opportunity to identify and address potential harmful effects (Asghari et al., 2020). This study aims to explore resident physicians’ experiences of the transition to pandemic care during the first wave of the COVID-19 pandemic in Sweden.

## 2. Materials and methods

This study evolved while analysing in-depth interviews with Swedish physicians in various positions about their experiences of working during the first wave of the COVID-19 pandemic (Jacobsson et al., 2022; Nilsson et al., 2022). In the analysing process, a pattern emerged in the interview material from resident physicians, which is the focus of this paper. Thus, in this study, we seek to gain a broader understanding of how these resident physicians‘in Sweden experienced the transition to pandemic care during the first wave of the COVID-19 pandemic.

### 2.1. Procedure

An advertisement was distributed on social media, in the journal of the Swedish Medical Association, and through the authors network. Resident physicians who were interested in participating were sent an invitation letter with information about the study.

A semi-structured interview guide was developed. The development of the interview guide was initiated in discussion between researchers and thereafter tested in five pilot interviews. After the pilots, the guide was fine-tuned before additional interviews were carried out. The guide included discussion areas with supporting questions and probes. Examples of discussion areas in the guide were: experiences of working during the transition to pandemic care, support, work and private life, quality and safe care, leadership, and views about the future.

Interviews occurred between June and October 2020 after the first wave of the pandemic. The interviews with resident physicians were conducted by the first and the second author through online video communication tools or in a place of the interviewee’s choice. The interviews took between 60 and 90 min. The interviews were recorded and transcribed verbatim by an external professional firm. Pauses and other verbal expressions were noted in the transcripts.

### 2.2. Participants

In total, 12 resident physicians agreed to be interviewed. The interviewees had completed an average of 3 years of their residency (1.5–4.5 years). The residents did their residency in medicine, infectious diseases, surgery, orthopaedics, obstetrics, anaesthesia, and family medicine. Six of the informants were women, and six were men. All participants had been working through the first wave of the pandemic.

### 2.3. Analysis

The empirical material was analysed using qualitative content analysis (Graneheim and Lundman, 2004; Graneheim et al., 2017). This method is appropriate for identifying empirically driven codes and categories. The qualitative content analysis moves from the manifest, close to the text descriptions and interpretations, to the latent content, more distant from the text but still close to the participant’s reality (Graneheim et al., 2017). The second author led the analytical procedure with support from the first author.

Initially, the full transcripts were read to get a sense of context. A basic decision was to select the units of analyses. We selected following units; emotional support, organisation of work, and instructional support. Within the three units, codes were identified and interpreted, and those with related meanings and shared characteristics were sorted into sub-categories. Similarities and differences across units and sub-categories were discussed between all authors, and sub-categories were then merged into categories. Finally, one theme unifying the latent content of the four categories was formulated through reflection and discussion. The analytical process following Graneheim and Lundman (2004) is inductive, moving back and forth in the analytical steps. The research team had different professions. One was a resident physician, one a specialist nurse and associate professor in occupational medicine, and one a social scientist and professor in health sciences. This gave various perspectives in the analyses and a holistic understanding of the transition to pandemic care.

### 2.4. Ethics

The project was undertaken according to research ethics guidelines. The study was ethically reviewed and approved by the Ethics Review Authority (ref: 2020-02433). The material was immediately anonymised to identify data in the transcriptions of the interviews. All data were properly stored according to the Swedish Act on Ethical Review of Research Involving Humans [SFS 2003:460 (2005)].

## 3. Results

The analysis resulted in one theme, four categories and seven sub-categories (Table 1). The theme identified in the material was *An impaired learning environment* which signifies the disruptions the resident physicians experienced during the first wave of the pandemic. The interviewed residents expressed in the interviews concern over the risk that their diploma of specialised doctor would be delayed, which will have implications both for themselves as physicians and for healthcare.

**Table 1:** Sub-categories, categories, and theme.

Sub-categories	Categories	Theme
Uncertainty and worry	Professional role insecurity	An impaired learning environment
Experience of isolation		
Feelings of exclusion	High expectations but little influence	
Expected to take on responsibility		
Missed training opportunities	Stagnant clinical development	
Peer support	Professional competence through experience	
Role expansion

My training will be prolonged. That’s the case for many other [residents]. It probably takes 6 months extra for me to become a specialist [receive a certificate of specialist expertise] because simply I cannot do my [required] training. […] But it will have implications on the healthcare services, there are delays for many resident physicians. [IP10].

The theme *An impaired learning environment* is signified by the categories Professional role insecurity, High expectations but little influence, Stagnant clinical development, and Professional growth through experience.

### 3.1. Professional role insecurity

The category *Professional role insecurity* concerns expressed uncertainty and worries as well as feelings of isolation during the initial phase of the COVID-19 pandemic by the interviewed resident physicians. The absence of knowledge created a vacuum in which uncertainty and worries grew. The resident physicians felt invisible, citing that they were working alone with few that understood what was happening. They also felt that expectations and circumstances changed from day to day or hour to hour. The interviewees described that they experienced a lack of leadership, policy, and information from management regarding how to proceed. The interviewees described experiences of a lack of clear directives from management regarding what applied “right now” both with regard to patient care but also directives concerning protective measures. This mainly concerned the work with COVID patients and logistics such as where meetings were to be held and which information channels were applied. Unclear directives made daily clinical work more difficult.

But then, what I would have liked, would probably have been … a little clearer decision-making, that it was not left to… to the colleague to try to figure out again and again, what to do and so on. [IP1].

The interviewees described the difficulties experienced in sorting through all the information and various directives that came about during the pandemic. Information could be communicated *via* various e-mails, websites, morning meetings, webinars, notes posted at the reception, etc. The quantity of information, the number of channels from which this information came, the unclear directives, and the lack of leadership meant that the resident physicians expressed a feeling of insecurity and frustration both in relation to the work and in relation to their training to become certified.

On the other hand, it was a bit frustrating when things changed from day to day, and you did not really get the communicated message of what was going on effectively. [IP8].

In the void of clear directives and routines, the resident physicians experienced feeling isolated. There was a lack of reflective discussion on moral and ethical aspects nor practical medical decisions. The interviewees describe that they felt they could not influence the activities during the pandemic. They expressed that many decisions which affected them were made without being consulted. For example, the emergency room was renovated and adapted to COVID patient care. Still, despite the emergency room being staffed chiefly by resident physicians, therefore a source of experience and knowledge, their opinions about what they felt was important for the improvement of care were not requested.

Yes, it was more or less like this that when you came in on a Monday, they were renovating, and no one knew what was going on or what was happening and how it would work and so on. And I think we probably had a lot, we as resident physicians who actually work there both day and night, probably had a lot of thoughts about what to do and how to control flows and so on and tried to think a little around that, and for a while, we were active and tried to access to the forums where decisions were made about this, but we received no response to [our inquires]. [IP8].

Feelings of isolation were also noted as one resident physician described the on-call consulting physicians were unexperienced with COVID patients, yet they had the role of support physicians to the resident physicians treating COVID patients.

### 3.2. High expectations but little influence

The category *High expectations and little influence* include the sub-categories Excluded and Expected to take on responsibility. The situation was somewhat contradicting or imbalanced. On the one hand, the resident physicians were excluded from important decisions, but on the other hand, these resident physicians were required to take on more responsibility than expected from a physician in training.

The resident physicians describe that they were excluded from the clinical work around patients. Medical decisions were made above their heads, and they had no way of influencing them. The interviewees felt ignored when making suggestions on patient treatments.

I had an attending physician that did not think we should be bothered with testing and protective equipment. And when we had inpatients, there were two older patients that were here with stated COVID. I do not know if both of them had pneumonia. At least one of the patients had pneumonia and bad lungs since before. I wanted to try to give the patient antibiotics besides oxygen, maybe it would have worked, and she could have gotten better. But he [the attending] just said no, we do not do that. I think that was hard. [IP12].

Some expressed frustration as they felt pushed away from medical procedures. For instance, there were fewer surgeries, and the surgeries conducted were done by attending physicians rather than resident physicians.

The inability to influence or affect medical decisions was also made clear in the resident physicians’ descriptions of how they were required to take increased responsibility for parts of the job that did not fall under their responsibility. They stated that there was an expectation to work longer shifts and extra on-call hours as well as make decisions that fell under the responsibility of the attending physician. As the principal care provider in the emergency room, the interviewees often had to answer questions about hospital management and take greater responsibility for the interns who worked there.

[Then] I was a little worried, when we would have that meeting about what we were going to do, because it was just me as [the only] doctor there, and then it was my managers and the managers from the emergency room, but the one who was medically responsible for the emergency room or for infection was not there. And then it was like “[IPs name] how do you think we should do?” And it felt like this; it is completely unreasonable that you gather these [qualified] people and then ask me, as the resident physician. [IP2].

### 3.3. Stagnant professional development

The interviewees described that they felt that their education stagnated when most of the training sessions did not take place. During the first wave of the pandemic, the specialty expertise courses were cancelled. External residency-terms, i.e., when resident physicians work, for a specific duration of time, in other specialty units, were withdrawn for some of the resident physicians. For others, they attended one of their external residency-terms during the pandemic but found themselves without supervision.

The interviewees described that besides the cancellation of the mandatory courses and external residency-terms, other training sessions did not occur. For instance, they said that they had less supervision and did not meet enough patients. The resident physicians, specifically in the operating specialities, expressed great frustration over stagnant development as a result of cancelled operations, external residencies and a reduced patient flow.

We have had much fewer elective surgeries, which has affected me quite a lot in my residency program, for what is to be operated on and who is to operate and so on, that a lot of such things have been cancelled, which has meant that we have had much fewer training opportunities. [IP1].

Yes, yes, but for me it became very obvious as I was about to enter an external resident term myself just then when the pandemic came. So, then it was a decision on the part of the region not to send resident physician away, you could apply for a special exemption, but everything felt so insecure. [IP5].

These cancelled learning activities contributed to frustrations and worries that they might not get their certificate of specialist expertise.

### 3.4. Professional competence through experience

Despite significant changes in the work with an impact on their professional development, there are positive aspects of their experiences of working during the COVID-19 pandemic. We identified two sub-categories that contributed to the residents’ growth as physicians: peer support and role expansion.

The support received from close colleagues is seen to have contributed to a positive environment and attitude to work, creating a sense of community.

But I think it [the pandemic] has had a positive effect on the sense of community and that you support and help each other when needed. I think that shows that we are a bit understaffed but still a well-knit team. [IP6].

Although many experienced a lack of leadership during the pandemic, many also described their immediate superior as crucial for information sorting, reconciliation, and reflection. Those with this experience expressed how important the managerial role has been during the pandemic. The support from the manager and colleagues is described as something good and important.

I think that both I and my other colleagues have been very pleased with the commitment of our boss and their drive to be quite early on things. [IP3].

The interviewees described a feeling of having been involved in something big, something they had not wanted to miss. They described that they experienced the work during the pandemic as something meaningful and that the pandemic was a training session.

I have gained more experience… or more confidence in my clinical skills […] more moral and ethical sentiments in relation to the patients and relatives that I meet. More experience! [IP9].

## 4. Discussion

This study explored how 12 resident physicians in Sweden experienced The transition to pandemic care during the first wave of the COVID-19 pandemic in Sweden.

To the best of our knowledge, this is among the few articles exploring how physicians-in-training experienced the pandemic impacted them. The analysis of the empirical material resulted in the theme, *An impaired learning environment*. The theme describes that the pandemic had a negative impact on the learning environment for resident physicians. In addition to performing clinical work, resident physicians must also acquire specialist competence in parallel, which imply meeting respective learning objectives. To do that, a quality learning environment is essential (Roff et al., 2005). The four categories give an in-depth to how the resident physicians experienced that the learning environment was impaired. These were: Professional role insecurity, High expectations but little influence, Stagnant clinical development, and Professional growth through experience.

The first wave of the COVID-19 pandemic choked healthcare services, and for the physicians working there, it was an intense period (Billings et al., 2021; Mohammadi et al., 2021; Jacobsson et al., 2022; Nilsson et al., 2022). The first wave of the pandemic is, therefore, of importance to study. Overall, the interviewees experienced a lack of leadership and guidance in everyday clinical practice. There was plenty of information which sometimes changed by the hour. As shown in previous research (Billings et al., 2021; Hertelendy et al., 2021; Mohammadi et al., 2021; Jacobsson et al., 2022; Nilsson et al., 2022), there was a lack of preparedness, and the leadership had little or no information about anything. This situation brought feelings of uncertainty and worry to the resident physicians interviewed in this study. The interviewees also expressed that the situation they were in was somewhat lonesome, that they had no one to ask, and that they were alone in decisions. Good supervision and a good relationship with the supervisor are preventative factors in stressful events such as the COVID-19 pandemic (Dyrbye et al., 2010, 2018; Abdelsattar et al., 2021). Previous research also shows that an imbalance between education and clinical work poses a risk of ill health among resident physicians (Lu et al., 2021).

The results from this study showed that during the pandemic, high demands and expectations were placed on the resident physicians to navigate the huge flow of information and on advanced clinical performance. Meanwhile, as high demands were placed on the resident physicians’ capacity, several experienced a lack of influence, and many decisions were made “over their heads.” Shapiro (2021) describe this imbalance between high expectations on the one hand and lack of influence on the other as residents “having all the responsibility of being full-grown ‘adult’ doctors, while at other times needing to be protected and/or controlled, enjoying very few privileges of autonomous physicians.” The pandemic has emphasised the dual role of resident physicians as both trainees and physicians (Shapiro, 2021). While increased responsibility can contribute to professional development, an inappropriate increase in responsibility can be detrimental to the resident physicians and lead to role confusion. In fact, previous research shows that inability to influence their work environment can contribute to fatigue among resident physicians (Mihailescu and Neiterman, 2019). High expectations of education and clinical development and lack of independence have also been shown to have a negative impact on resident physicians’ mental health (Dyrbye and Shanafelt, 2016). Discrepancies and disparities in role perception between expectations, responsibility, and influence that affect residents negatively, particularly during crises, need to be a central aspect of any contingency plans.

The study shows that during the pandemic, many situations arose where essential pandemic care needed to be prioritised before educational elements. This stagnant clinical development has also been described in other studies (Dedeilia et al., 2020; Abdelsattar et al., 2021; Shapiro, 2021; Wådell et al., 2022). Although scheduled educational courses and teaching opportunities can be adjusted to online, hands-on training and surgery cannot (Wådell et al., 2022).

Despite an impaired learning environment, the resident physicians described that their experiences contributed to professional development. However, as Shapiro (2021) states, although resident physicians have developed the clinical skills necessary in crisis, there might still be developmental delays in their professional identity in their chosen specialty. Nevertheless, resident physicians are often those that receive patients at the emergency units. The resident physicians interviewed described being sent to the front line. As such, their knowledge and experience of the COVID-19 pandemic must be valued and in consideration for future pandemics.

The impaired learning environment now needs healing. The experiences of work and specialist training by resident physicians during the first wave of the COVID-19 pandemic can have a negative impact on their mental health (Dyrbye and Shanafelt, 2016; Dyrbye et al., 2018; Mihailescu and Neiterman, 2019; Lu et al., 2021). Previous studies show that a fractured learning environment is a risk factor for burnout (van Vendeloo et al., 2018; Lu et al., 2021). Meanwhile, a recent Swedish study showed that the prevalence of burnout among resident physicians was as high as 6.8%, which is higher than among attending and consulting physicians (Hagqvist et al., 2022). While poor learning environments can be detrimental to resident physicians’ mental health, a quality learning environment can have the opposite effect (Ironside et al., 2019). It is evident that employers now must ensure a quality learning environment for resident physicians during a crisis (Abdelsattar et al., 2021; Wådell et al., 2022).

### 4.1. Methodological discussion

We analysed transcripts of interviews with 12 resident physicians. The data material analysed represent their experiences. The interviews were done during the first wave of the pandemic and the physicians might have been anxious in a way that affected their answers. The physician’s were although very positive to participate in the study and conveyed that they were glad to share their experiences. We considered that we had a rich and saturated material (Fusch and Ness, 2015). Saturation was gained upon ceasing to result in new information related to the categories (Patton, 2002). To ensure thoroughness, the authors discussed the empirical material during the analytical process and cross-checked the data and interpretations (Cypress, 2017). Dependability was achieved through the found methodological experience among the authors and the authors’ understanding of the healthcare context. The results should not be generalised, but it is likely that they can be relevant in other settings (Polit and Beck, 2004) where resident physicians have been working during the pandemic.

## 5. Conclusion

This study shows that resident physicians’ learning environment was affected by the COVID-19 pandemic. As healthcare must now transition from pandemic care to paying off the associated debt created by that care, clinics and regions should manage resources to prioritise the resident physicians’ training and thereby ensure a good learning environment. Furthermore, contingency plans should include strategies for how to secure resident physicians learning environment, and to decrease the need for a prolonged education while saving lives. It is important the resident physicians are included and that they have access to supervision and support.

## Data availability statement

The raw data supporting the conclusions of this article will be made available by the authors, without undue reservation.

## Ethics statement

The study was approved by the Swedish Ethical Review Authority (2020-02433). The project was undertaken according to research ethics guidelines and written informed consent was obtained from all participants at the start of the interviews. The resident physicians were told that their participation was voluntary and that they could withdraw from the study at any time. All the participants gave oral and written consent.

## Author contributions

EB designed the study and the project and conducted four interviews. KH conducted eight interviews and had the main responsibility of analysing the empirical material with support from EB. KH wrote the first draft of the manuscript and EB continued. BL gave critical comments during the analytical process and on the manuscript. All authors contributed to the article and approved the submitted version.

## Funding

This study was funded by Swedish Research Council for Health, working life and welfare (2019-00311) and Region Stockholm (20191179).

## Conflict of interest

The authors declare that the research was conducted in the absence of any commercial or financial relationships that could be construed as a potential conflict of interest.

## Publisher’s note

All claims expressed in this article are solely those of the authors and do not necessarily represent those of their affiliated organizations, or those of the publisher, the editors and the reviewers. Any product that may be evaluated in this article, or claim that may be made by its manufacturer, is not guaranteed or endorsed by the publisher.
